# Antioxidant, photoprotective and inhibitory activity of tyrosinase in extracts of *Dalbergia ecastaphyllum*

**DOI:** 10.1371/journal.pone.0207510

**Published:** 2018-11-30

**Authors:** Daniel Vieira de Morais, Maria Angélica Pereira de Carvalho Costa, Marivalda Figueredo Santa Bárbara, Fabiane de Lima Silva, Manuela M. Moreira, Cristina Delerue-Mato, Luís Avelino Guimarães Dias, Maria Leticia Miranda Estevinho, Carlos Alfredo Lopes de Carvalho

**Affiliations:** 1 Centre for Agricultural, Environmental and Biological Sciences, Federal University of Recôncavo of Bahia/UFRB, University Campus, Cruz das Almas, Bahia, Brazil; 2 REQUIMTE/LAQV, Superior Institute of Engineering of Porto, Porto, Portugal; 3 Mountain Research Center, Polytechnic Institute of Bragança, Campus Santa Apolónia, Bragança, Portugal; National University Singapore Yong Loo Lin School of Medicine, SINGAPORE

## Abstract

*Dalbergia ecastaphyllum* is a native Brazil plant with importance for beekeeping, and widely used in folk medicine. For the first time, the extracts of this plant were assessed for the presence of hydrophilic and lipophilic antioxidants, as well as inhibition of tyrosinase, free radicals scavenging and sunscreen protection. The antioxidant activity was evaluated by free radical scavenging (DPPH) and β-carotene bleaching assay. The tyrosinase inhibitory activity was evaluated and calculated the EC50. The photoprotective activity was measured using different concentrations of *D*. *ecastaphyllum* extracts. The Sun Protection Factor (SPF) of the samples was higher than 6, and the sample from Ilhéus showed the most pronounced photoprotective effect. Sample from Canavieiras presented the highest antioxidant activity by free radical scavenging DPPH and β-carotene bleaching method, with 92.41% and 48.34%, respectively. All samples inhibited the tyrosinase, especially the sample from Prado that was most effective (124.62 μg.mL^-1^). Significant negative correlation was found between flavonoid contents and inhibition of tyrosinase. The overall results provide relevant information about the *Dalbergia ecastaphyllum* species, indicating as potential material to cosmetic and pharmaceutical industry.

## Introduction

The formation of free radicals is a continuous and physiological process with important biological functions [1], which include cellular respiration, important role in the immune system and phagocytosis, cell growth regulation and synthesis of biological substances. However, when they are produced and accumulated in excess in the body, free radicals cause oxidative stress, a phenomenon that can cause various chronic and degenerative diseases, particularly, the metabolic syndrome, diabetes, various types of cancer and the Alzheimer’s disease [2]. Free radicals are also involved in the aging process and in some acute pathologies (trauma, stroke) [3].

Some examples of free radicals are: hydroxyl radical–OH., superoxide anion (O2.-), peroxyl-radical ROO., alcoxyl radical–RO and nitric oxide–NO. Among these free radicals, OH. and O_2_ have the greatest biological importance [4].

Antioxidant compounds can be used to control the production of these substances, which may delay or inhibit the lesions caused by free radicals. These compounds include vitamins, minerals, natural pigments and other plant compounds and enzymes. Thus, studies that identify natural resources able to act as antioxidant agents are extremely important. These antioxidants can be endogenous or come from a diet and other sources such as tocopherols (vitamin E), ascorbic acid (vitamin C), polyphenols, selenium and carotenoids [5, 6].

Tyrosinase is a multifunctional enzyme widely distributed in nature (bacteria, fungi, plants, and animals) [7]. The main substrate of this enzyme in mammals is the amino acid tyrosine, which, after several chemical and enzymatic reactions, polymerizes and forms melanin. Melanin is a heterogeneous biopolymer that plays an important role in protection against the harmful effects of ultraviolet radiation (UV) on human skin and it is also responsible for the color of skin, eyes and hair [8, 9]. However, when accumulated in excess, melanin results in hyperpigmentation disorders such as lentigo, naevus, freckles, age spots, chloasma and melanoma. Pigmentary disorders resulting from changes in the level of tyrosinase activity is the third cause of melanogenesis [10]. Thus, understanding the regulatory activity of this enzyme is of relevant interest to the pharmaceutical and cosmetic industries. To fight these types of disorders, the so-called “tyrosinase inhibitors”, mainly, hydroquinone, Kojic acid and retinoids are used; however, these inhibitors have been reported to cause adverse effects such as cytotoxicity and mutagenicity, limiting their use in humans [11, 12].

Therefore, research on new agents, particularly natural products that inhibit the tyrosinase activity and that are effective in the hyperpigmentation treatment, is of utmost relevance.

Tyrosinase is also reported in studies on the Parkinson’s disease, a progressive neurodegenerative disorder characterized by selective degeneration of dopamine. When produced in excess, tyrosinase is able to increase intracellular dopamine production, followed by the induction of melanin formation, causing cell death [13].

*Dalbergia ecastaphyllum* (L.) Taub is a plant species found in Brazil with great ecological and economic importance, especially concerning apiculture pasture and red propolis production. Unfortunately, over the years, populations of *D*. *ecastaphyllum* have greatly reduced due to anthropic actions through burning and deforestation.

In this work, for the first time, hydro-ethanol extracts of *D*. *ecastaphyllum* (L) Taub., from Brazil, were investigated in terms of contents of total phenols, flavonoids, carotenoids and chlorophylls, as well as antioxidant, photoprotective and inhibitory activity of tyrosinase.

## Material and methods

No specific permits were required for the field studies. The collections were carried out in public areas, where no authorization was required for the execution of the work. The fields did not involve endangered or protected species.

### Plant materials

Leaves of *D*. *ecastaphyllum* were collected in seven municipalities (Table 1) in the State of Bahia, Brazil, in October 2014. The samples were oven-dried in air circulation (40°C) for 72 h, ground in grinder, stored in closed polycarbonate flasks, kept under room temperature and protected from light.

**Table 1:** Geographic data of *Dalbergia ecastaphyllum* collected from seven municipalities in the state of Bahia.

Sample code	Municipalities	Coordinates	
		Latitude	Longitude
**T1**	Itaparica	12° 53’ 18” S	38° 40’ 43” W
**T2**	Vera Cruz	13° 5’ 60" S	38° 45’ 00” W
**T3**	Nova Viçosa	17° 57' 40.8" S	39° 27' 53.8" W
**T4**	Prado	17° 06' 11.8" S	39° 15' 54.9" W
**T5**	Caravelas	17° 43' 57.4" S	39° 15' 54.9" W
**T6**	Ilhéus	15° 40’ 38” S	38° 56’ 42” W
**T7**	Canavieiras	14° 47’ 36” S	39° 2’ 46” W

### Power calculation

A power calculation was conducted to determine how many samples should be needed to collect in each place. The test was performed using the BioEstat 5.0 [14] software by means of the size test of ANOVA (analysis of variance) with significance level with α = 0.05 (two-sided) and power of the statistical test 80%. Minimum difference between treatments: 80; Standard error deviation: 10; number of treatments: 7; number of replicates per treatment: 4.

### Preparation of the extracts

For the preparation of hydro-ethanol extracts, 15 g of each sample were macerated in hydro-ethanol solution (water/ethanol ratio: 3:7 v/v) under constant stirring at room temperature for 14 days. Subsequently, the extracts were filtered in filter paper and rotary evaporators (IKA RV-USA) at 40°C until total evaporation of the solvent. The dried extracts were stored in closed tubes and kept under room temperature in the absence of light.

### Determining carotenoids and chlorophylls

The compounds β-carotene, lycopene, and chlorophylls *a* and *b* were determined using an established procedure [15], with some modifications. For that purpose, 150 mg of the powdered samples were stirred in 10 mL of acetone/hexane (4:6 v/v) (Synth-BRA) in the Vortex for 1 min and filtered through filter paper. Absorbance (A) was measured in a spectrophotometer (Unican Helios) at wavelengths 453, 505, 645 and 663 nm.

The quantification was carried out according to the following equations:

$$\beta ‑carotene\;\left(mg/100\;mL\right)=0.216\times A_{663}-1.220\times A_{645}-0.304\times A_{505}+0.452\times A_{453};$$

$$Lycopene\;\left(mg/100\;mL\right)=-0.0458\times A_{663}+0.204\times A_{645}-0.304\times A_{505}+0.452\times A_{453};$$

$$Chlorophyll\;a\;\left(mg/100\;mL\right)=0.999\times A_{663}-0.0989\times A_{645};$$

$$Chlorophyll\;b\;(mg/100\;mL)=-0.328\times A_{663}+1.77\times A_{645}.$$

The results were expressed in mg per 100 g of dry mass.

### Determining total phenolic and flavonoids content

The total phenolic (TP) were estimated using a published method [16] with some modifications. In test tubes, an aliquot of 0.5 mL of extract solution (0.2 mg/mL) was mixed with reagent Folin-Ciocalteu (2.5 mL, Dinâmica-BRA) previously diluted in water (1:10 v/v) and 2.0 mL of sodium carbonate (7.5%, Dinâmica-BRA). After shaking, the mixture was left to rest for 30 min at 40°C. Subsequently, absorbance was measured using a UV-Vis spectrophotometer (Unican Helios) at 765 nm. Gallic acid (Dinâmica-BRA) was used to obtain the calibration curve (concentration levels ranged from 0.15 to 0.93 μg/mL). The results were expressed in mg of Gallic acid equivalents (GAE) per g of dry weight.

The quantification of total flavonoids (TFL) was estimated as described previously by Zhishen et al. [17]. We it was blended 0.5 mL of the extract solution (55 μg/mL) with 2 mL of distilled water and 0.15 mL of NaNO_2_ solution (5%, Chemco-BRA). After the mixture was shaken and left to rest for 6 min, 0.15 mL of a solution of AlCl_3_ (10%, Neon-BRA) was added. Again, after shaking, the mixture was left to rest for 6 min and 2 mL of NaOH solution (4%, Vetec-BRA) and distilled water were added to reach a final volume of 5 mL. Absorbance of the final mixture, after 15 min of rest, was measured at 510 nm. Quercetin (Sigma Aldrich—USA) was used to construct the calibration curve (concentration levels ranged from 0.03 to 0.26 mM) and the results were expressed in mg quercetin equivalents (QE) per gram of dry weight.

### HPLC-PDA analysis of phenolic compounds

The identification and quantification of phenolic compounds was performed according to the method previously described by Rubilar et al. [18] *D*. *ecastaphyllum* extracts (20 μL) were injected into a HPLC system (Shimadzu Corporation, Kyoto, Japan) equipped with a LC-20AD prominence pump, a DGU-20AS prominence degasser, a CTO-10AS VP column oven, a SIL-20A HT prominence autosampler, and a SPD-M20A photodiode array detector (PAD). Separations were realized at 25°C on a *Phenomenex* Gemini C_18_ column (250 mm x 4.6 mm, 5 μm) and the chromatograms were recorded at 280, 320 and 360 nm depending from the phenolic compound maximum wavelength. Phenolic compounds were analyzed using a gradient elution at 1.0 mL/min with the following program: 85% B in 0 min, from 85% to 70% B in 20 min, from 70% to 55% B in 20 min, from 55% to 50% B in 5 min, from 50% to 45% B in 5 min, from 45% to 30% B in 15 min, from 30% to 0% B in 10 min, followed by 100% A for 5 min and back to 85% B in 10 min and 10 min of reconditioning before the next injection. The mobile phase was composed by methanol (solvent A) and water (solvent B) both with 0.1% formic acid. Phenolic compound identification was carried out by comparing UV absorption spectra and retention time of each compound with those of pure standards injected in the same conditions. For the quantification of phenolics, different concentrations of each standard were prepared from the respective stock solution (2000 mg/L in methanol), and the results were expressed as milligrams of compound per liter (mg/L).

### Antioxidant activity

#### Free radical scavenging activity of 2.2-*diphenyl*-1-picrilhidrazil (DPPH)

This procedure was performed using the method developed by Alencar et al. [19]. Sample solutions were prepared by adding 3 mL of ethanol and 0.3 mL of an ethanol solution containing radical DPPH (0.5 mM) to 0.5 mL of extracts under different concentrations (6, 4, 8, 16, 32, 48, 64 and 80 μg/mL). Afterward, the mixture was left to rest for 60 min in the dark. The DPPH radical reduction was determined by measuring absorbance at 517 nm. The antioxidant activity was calculated by the absorbance decline rate of the DPPH solution/samples in relation to the control solution (DPPH in ethanol, Sigma Aldrich -USA), according to the equation:

$$percentage\;of\;antioxidant\;activity=\left(1‑\left(Abs_{sample}/Abs_{control}\right)\right)*100,$$

Where: Abs_sample_ is the absorbance the DPPH sample solution and Abs_control_ is the absorbance of the control solution (DPPH in ethanol). The concentration (μg/mL) of extract needed to scavenge 50% of the initial concentration of DPPH (EC50) was calculated. Quercetin was used as positive control.

#### Self-oxidation of the β-carotene system/linoleic acid

This test was performed using an established procedure described by Ahn et al. [20], with some modifications. A solution of β-carotene (Sigma Aldrich—USA) was prepared dissolving this compound (5 mg) in chloroform (25 mL, Anidrol-BRA). It was transferred 4 mL of this solution, 80 μL of linoleic acid (Sigma Aldrich-USA) and 800 μL of Tween (Sigma Aldrich—USA) were added into a round-bottom flask. Next, this emulsion was evaporated at 40°C under vacuum to remove chloroform. It was transferred 4.8 mL aliquots of this emulsion to test tubes containing 0.2 mL of extract (80 μg/mL). Shortly after the addition of the emulsion, the aliquots were shaken and the time zero of absorbance was determined at 470 nm. Later, the aliquots were incubated in water bath at 55°C for 2 h. Afterward, absorbance was again determined at 470 nm.

It was prepared a bleach with extraction solvent (ethanol) to replace the extract solution. The antioxidant activity (AA) was expressed by percent inhibition in relation to control (emulsion + ethanol) after 120 minutes of incubation according to equation:

$$AA=\left(RDC_{control}-RDA_{sample}\right)/RDC_{control}*100,$$

Where RDC_control_ [= ln (a/b)/120] is the percentage of β-carotene whitening without the antioxidant presence, “In” is the natural logarithm, “a” is the absorbance in time 0, “b” is the absorbance after 120 min; and, DRA_sample_ [= ln (a / b) / 120] is the percentage of β-carotene bleaching with the antioxidant presence.

### Tyrosinase inhibitory activity

The tyrosinase inhibitory activity was determined according to the modified method using L-3.4-dihydroxyphenylalanine (L-DOPA) as substrate, as previously described [21], with some modifications. An aliquot of 25 μL of extract solution (1 mg/mL) was mixed with tyrosinase (40 μL, Sigma Aldrich- USA) and a buffering solution (100 μL, pH 6.8) in 96 wells of microplates (Multiskan GO Microplate Spectrophotometer–Finland) and incubated for 15 min at 25°C. The reaction was initiated with the addition of L-DOPA (40 μL, Sigma Aldrich—USA). Similarly, a bleach was prepared by adding the extract solution to all reagents except for the enzyme solution (tyrosinase). The sample absorbance and bleaching (Kojic acid, Sigma Aldrich—USA) were read at 492 nm after incubation at 25°C. The results were compared with the control (DMSO). The tyrosinase inhibitory activity was calculated based on the formula:

Tyrosinaseinhibitoryactivity%=[A0‑A1/A0]x100

Where: A0 is the absorbance at 492 nm with DMSO, replacing the sample, and A1 is the absorbance at 492 nm with the sample under analysis.

### *In vitro* photoprotective activity

#### Determining the maximum wavelength (ƛ_max_)

The maximum wavelength (ƛ_max_) was determined using the established procedure [22]. Dried extracts were diluted in ethanol (Synth-BRA) at a concentration 100 μg.mL^-1^ and scanning was performed using a spectrophotometer at wavelengths between 260 and 400 nm.

#### *In vitro* determination of the Sun Protection Factor (SPF)

To determine the sun protection factor (SPF), dried extracts were diluted in ethanol (32, 48, 64, 80 and 100 μg/mL) and absorbance was read between wavelengths 290 and 320 nm. The average SPF was calculated according to the equation [23]:

$${\mathrm{SPF}}_{\mathrm{spectrophotometric}}=\mathrm{CF}\times \sum_{290}^{320}EE(\lambda )\times I(\lambda )\times A(\lambda )$$

Where: CF is the correction factor (equal to 10), EE(**λ**) is the erythematogenic effect of wavelength (**λ**) radiation; I(**λ**) is the sunlight intensity at wavelength **λ**; A(**λ**) is the spectrophotometric reading absorbance of the preparation solution at wavelength **λ**.

### Statistical analyses

The tests of each sample were carried out in triplicate. The results were expressed as mean value ± standard deviation (SD).

Data were analyzed by the analysis of variance (ANOVA) considering the completely randomized design and, when significant, the means of the treatments were compared using the Tukey test. The normality of the distribution were performed for all data collected using the Shapiro–Wilk test. All tests were evaluated considering the significance level p < of 0.05. The multivariate analyses were performed: (i) principal components analysis (PCA) in order to better understand the relationship between antioxidant compounds and the inhibitory characteristic of tyrosinase; (ii) canonical correlation analysis (CVA) was performed as supplementary analysis to verify associations between groups of characteristics X (antioxidant compounds) and characteristics Y (antioxidant methods). For the PCA and CVA analyses, the phenotypic correlation matrix was used. All analyses were performed using the program R version 3.3.0 [24].

## Results and discussion

The objective of this study was to evaluate the tyrosinase inhibition as well as the antioxidant and photoprotective activity of *D*. *ecastaphyllum* extracts.

The yield obtained by the extraction for each treatment was as follow: T1 = 18%; T2 = 20,56%; T3 = 25,13%; T4 = 28,19%; T5 = 23, 68%; T6 = 20,88% and T7 = 25,65%.

### Chemical characterization of *D*. *ecastaphyllum* extracts

The results obtained for the contents of pigments lipophilic antioxidants are summarized in Table 2.

**Table 2:** Composition of lipophilic antioxidants (mg/100 g of dry mass) of samples of *Dalbergia ecastaphyllum*.

Sample code	β-carotene	Lycopene	Chlorophyll *a*	Chlorophyll *b*
**T1**	0.14 ± 0.01 *a*	0.41 ± 0.01 *b*	0.66 ±0.01 *d*	0.33 ± 0.01 *bc*
**T2**	0.08 ± 0.01 *b*	0.35 ±0.01 *cd*	0.58 ± 0.01 *ef*	0.28 ± 0.03 *c*
**T3**	0.08 ± 0.01 *b*	0.40 ± 0.01 *b*	0.72 ± 0.01 *c*	0.39 ± 0.01 *ab*
**T4**	0.06 ± 0.01 *cb*	0.36 ± 0.01 *c*	0.61 ± 0.02 *ed*	0.35 ± 0.02 *bc*
**T5**	0.14 ±0.01 *a*	0.40 ± 0.01 *b*	0.54 ± 0.02 *f*	0.30 ± 0.02 *c*
**T6**	0.03 ± 0.01 *c*	0.33 ± 0.01 *d*	0.84 ± 0.02 *b*	0.39 ± 0.03 *ab*
**T7**	0.16 ± 0.01 *a*	0.50 ± 0.01 *a*	0.97 ± 0.02 *a*	0.41 ± 0.02 *a*
**p-value**	3.00	3.00	3.00	3.00

Means followed by the same letter within a column do not differ at Tukey’s test at p-value *<* 0.05.

The β-carotene content ranged from 0.03 ± 0.01 to 0.14 ± 0.01 mg/100 g of dry mass. The values found for lycopene were higher and ranged from 0.33 ± 0.01 to 0.50 ± 0.01 mg/100 g of dry mass. The highest level was obtained for the sample T7, while T1, T3 and T5 samples have intermediate and statistically similar concentrations, followed by the other samples, with low and comparable levels. Plants and some microorganisms can synthesize carotenoids and chlorophylls, while animals and humans are incapable [25]. The compounds are considered natural pigments that provide protection against oxidative stress and thus play an important antioxidant role [26].

Among the lipophilic compounds evaluated, chlorophyll *a* presented a greater content and, again, mainly in sample T7, which had a higher average of 0.97 ± 0.02 mg/100 g of dry mass. Also, statistically this parameter showed higher variability in its results. The T3, T6 and T7 samples presented the highest contents of chlorophyll *b*, compared to the other samples, having the T2 and T5 samples, the lower levels.

The results for the total phenolic (TP) and flavonoids content (TFL) are shown in Table 3. For TP in general, there were significant differences among the samples studied, ranging from 297.89 ± 4.54 to 378.43 ± 10.51 mg GAE/g of dry extract. The highest values were observed in samples T5 and T3, 378.43 ± 10.51 and 377.17 ± 10.50 mg GAE/g of dry extract, respectively. Due to the obtained experimental errors, the other samples present statistically similar mean values. For TFL, the highest content was observed in sample T6 (28.59 ± 2.10 mg EQ/g of dry extract) whose value differed statistically from the others, ranging between 10 and 20 mg EQ/g of dry extract.

**Table 3:** Total phenolic and flavonoids contents in hydro-ethanol extracts of *Dalbergia ecastaphyllum*.

Sample code	Total Phenols (TP) (mg GAE/g dry weight)	Total flavonoids (TFL) (mg QE/g dry weight)
**T1**	298 ± 4 *b*	11 ± 3 *c*
**T2**	336 ± 8 *ab*	13.2 ± 0.6 *cb*
**T3**	377 ± 10 *a*	19.5 ± 0.3 *b*
**T4**	302 ± 7 *ab*	16 ± 3 *cb*
**T5**	378 ± 10 *a*	19 ± 3 *b*
**T6**	337 ± 4 *ab*	28 ± 2 *a*
**T7**	351 ± 4 *ab*	13 ± 3 *cb*
**p-value**	3.00	3.00

Means followed by the same letter within a column do not differ at Tukey’s test at p-value *<* 0.05.

Flavonoids are considered hydrophilic antioxidants that act as antimicrobials, photoreceptors and are responsible for important functions in plants such as pigmentation, UV protection and defense against pathogens [27, 28]. In human health, these phenolic compounds have potential effect beneficial due to their capacity scavenging of free radicals that are related to several diseases such as cancer and cardiovascular diseases [29].

This is the first time that phenolic compounds of *D*. *ecastaphyllum* leaf extracts from South America have been determined.

In our present study, eleven phenolic compounds were identified and quantified by HPLC-PAD analysis (Table 4). Five belong to phenolic acids group: caffeic, sinapic, vanillic, protocatechuic and β-resorcylic acids. Where, the first two are hydroxycinnamic acid derivatives and the others are hydroxybenzoic acid derivatives. These substances are widely present in fruits, vegetables and nuts. Its potential bioactivity has been reported for its diverse properties as antioxidant, anthelmintic and antimicrobial [30]. The others six phenolic compounds identified belong to flavonoids group including four different classes: flavanols (catechin and epicatechin), flavonols (quercentin), flavanones (naringin and naringenin) and flavones (rutin). Flavonoids are substances widespread found in plants known to be potent antioxidants with remarkable effects against diverse diseases such as cancer, neurodegenerative or cardiovascular disease [31].

**Table 4:** Phenolic components (mg/L) in hydro-ethanol extracts of *Dalbergia ecastaphyllum* (mean ± SD) by HPLC-PAD.

Compounds	Retention time (min)	Content (mg/L)						
		T1	T2	T3	T4	T5	T6	T7
**Caffeic acid**	23.775	nd	nd	0.26 ± 0.01	0.29 ± 0.01	nd	nd	nd
**Catechin**	17.35	12.49 ± 0.62	6.62 ± 0.33	28.03 ± 1.40	19.95 ± 1.00	1.56 ± 0.08	3.68 ± 0.18	26.32 ± 1.32
**Epicatechin**	24.419	0.80 ± 0.04	0.65 ± 0.03	1.35 ± 0.07	0.91 ± 0.05	nd	nd	0.91 ± 0.05
**Naringenin**	54.98	0.64 ± 0.03	0.36 ± 0.02	1.39 ± 0.07	1.23 ± 0.06	0.26 ± 0.01	0.77 ± 0.04	1.39 ± 0.07
**Naringin**	41.85	1.99 ± 0.10	1.67 ± 0.08	1.70 ± 0.08	1.28 ± 0.06	1.28 ± 0.06	3.72 ± 0.19	3.73 ± 0.19
**Protocatechuic acid**	12.12	1.65 ± 0.08	1.13 ± 0.06	2.53 ± 0.13	1.37 ± 0.07	2.34 ± 0.12	1.60 ± 0.08	1.66 ± 0.08
**Quercetin**	58.658	nd	nd	0.93 ± 0.05	0.98 ± 0.05	nd	nd	nd
**Rutin**	44.68	10.49 ± 0.52	4.83 ± 0.24	18.52 ± 0.93	17.98 ± 0.90	11.04 ± 0.55	8.00 ± 0.40	20.23 ± 1.01
**Sinapic acid**	35.90	0.63 ± 0.03	0.28 ± 0.01	0.95 ± 0.05	0.94 ± 0.05	0.65 ± 0.03	0.50 ± 0.02	1.28 ± 0.06
**Vanillic acid**	22.88	1.35 ± 0.07	0.90 ± 0.04	2.19 ± 0.11	1.45 ± 0.07	0.99 ± 0.05	1.42 ± 0.07	2.07 ± 0.10
**β -resorcylic acid**	25.91	10.37 ± 0.52	6.72 ± 0.34	14.54 ± 0.73	22.12 ± 1.11	8.66 ± 0.43	8.03 ± 0.40	20.72 ± 1.04

nd: not detectable.

The major phenolic compound detected was catechin (1.56 ± 0.08 to 28.03 ± 1.40), rutin (4.83 ± 0.24 to 20.23 ± 1.01) and β -resorcylic acid (6.72 ± 0.34 to 22.12 ± 1.11). Changes in content of phenolic compound observed to *D*. *ecastaphyllum* leaf extract can be attributed to the sample origin. It is important to highlight that leaf age, different environmental conditions may affect the content and biosynthesis of phenolics in leaves [32].

### Antioxidant activity

The use of synthetic antioxidants is associated to damage to health, and because of this, a trend for utilization of antioxidants from natural sources, such as plants, has been developed [33]. The values of the antioxidant activity were obtained for the same extract concentration (80 μg/mL) by two different methods: free radical scavenging capacity (DPPH), which is a test based on the reaction transfer of hydrogen atoms, and inhibition of β-carotene bleaching, which is based on the electron transfer reaction [34].

Regarding the antioxidant activity by the DPPH method, sample T7 showed the greatest antioxidant capacity (lower EC50 values), 28 μg/mL, followed by T5, with 30.20 μg/mL (Table 5).

**Table 5:** Hydrophilic antioxidants and antioxidant activity of hydro-ethanol extracts of *Dalbergia ecastaphyllum*.

Sample code	Free radical scavenging (DPPH) EC 50 μg/mL	β-carotene bleaching assay (%)
**T1**	47.0 ± 0.8 *a*	22 ± 1 *e*
**T2**	35.8 ± 0.9 *ab*	26 ± 1 *d*
**T3**	40.1 ± 0.6 *ab*	13 ± 1 *f*
**T4**	34.0 ± 1.0 *abc*	8 ± 2 *g*
**T5**	30.2 ± 0.8 *bc*	7.4 ± 0.3 *g*
**T6**	40 ± 2.0 *ab*	40 ± 1 *c*
**T7**	28.0 ± 0.6 *bc*	48 ± 1 *b*
**Gallic acid**	18 ± 3 *c*	
**Quercetin**	34 ± 1 *abc*	
**BHA**		88. 3 ± 0.8 *a*
**p-value**	3.00	3.00

Means followed by the same letter within a column do not differ at Tukey’s test at p-value *<* 0.05.

These results show that no relevant differences were obtained in the samples antioxidant activity. In the inhibition test of β-carotene bleaching (Table 5), by descending order, the antioxidant activity of the samples was: T5 > T4 > T3 > T1 > T2 > T6 > T7.

The inhibition percentage evaluated by the inhibition test of β-carotene bleaching was lower than that determined by the DPPH method. The antioxidant activity quantified by the first methodology was, for most samples, identical to that obtained with quercetin, suggesting a high antioxidant potential of *D*. *ecastaphyllum* extracts, although not comparable with the antioxidant activity of gallic acid.

Interestingly, the two methods gave different results for all the samples since there was no correlation found between them, which can be explained by the different chemical mechanisms of reaction of these two methodologies and, therefore, with different reaction affinities to chemical compounds in the extract. In addition, individual antioxidant compounds do not necessarily reflect the total antioxidant capacity, as there can exist synergistic or antagonistic interactions between the different compounds [35].

#### Tyrosinase inhibitory activity

The enzyme tyrosinase is considered a key element in melanin synthesis [36].This enzyme converts L-tyrosine to L-DOPA (L-3.4-dihydroxyphenylalanine), which is subsequently oxidized to L-DOPA, originating dopachrome [37]. Tyrosinase inhibitors are used both in medicine, in hyperpigmentation treatments, and in cosmetics [38].

The results obtained in this study show that extracts of *Dalbergia ecastaphyllum* inhibited the tyrosinase activity (Table 6). The most pronounced inhibition was observed in sample T5, which can be inferred to be more dependent on the profile of phenolic compounds present in its composition than its total concentration. The literature reports that these compounds are responsible mainly for the inhibition of tyrosinase activity [39].

**Table 6:** Tyrosinase inhibitory activity of hydro-ethanol extracts of *Dalbergia ecastaphyllum*.

Sample code	Tyrosinase inhibition EC50 μg/mL
**T1**	184 ± 3 *c*
**T2**	472 ± 4 *a*
**T3**	172 ± 4 *d*
**T4**	148 ± 2 *e*
**T5**	125 ± 4 *f*
**T6**	202 ± 2 *b*
**T7**	463 ± 6 *a*
**Acarbose**	5.9 ± 0.3 *g*
**p-value**	3.00

Means followed by the same letter within a column do not differ at Tukey’s test at p-value *<* 0.05.

The inhibition induced by Kojic acid was greater than in the samples studied. Overall, significant differences in EC50 values among all the samples studied were found, with exception of T7 and T2 samples that also presented high values in tyrosinase activity. *D*. *ecastaphyllum* extracts used in this study inhibited more significantly the tyrosinase activity than extracts of *Alchemilla vulgaris* and *Filipendula ulmaria* [40].

### Photoprotective activity

**Fig 1:**
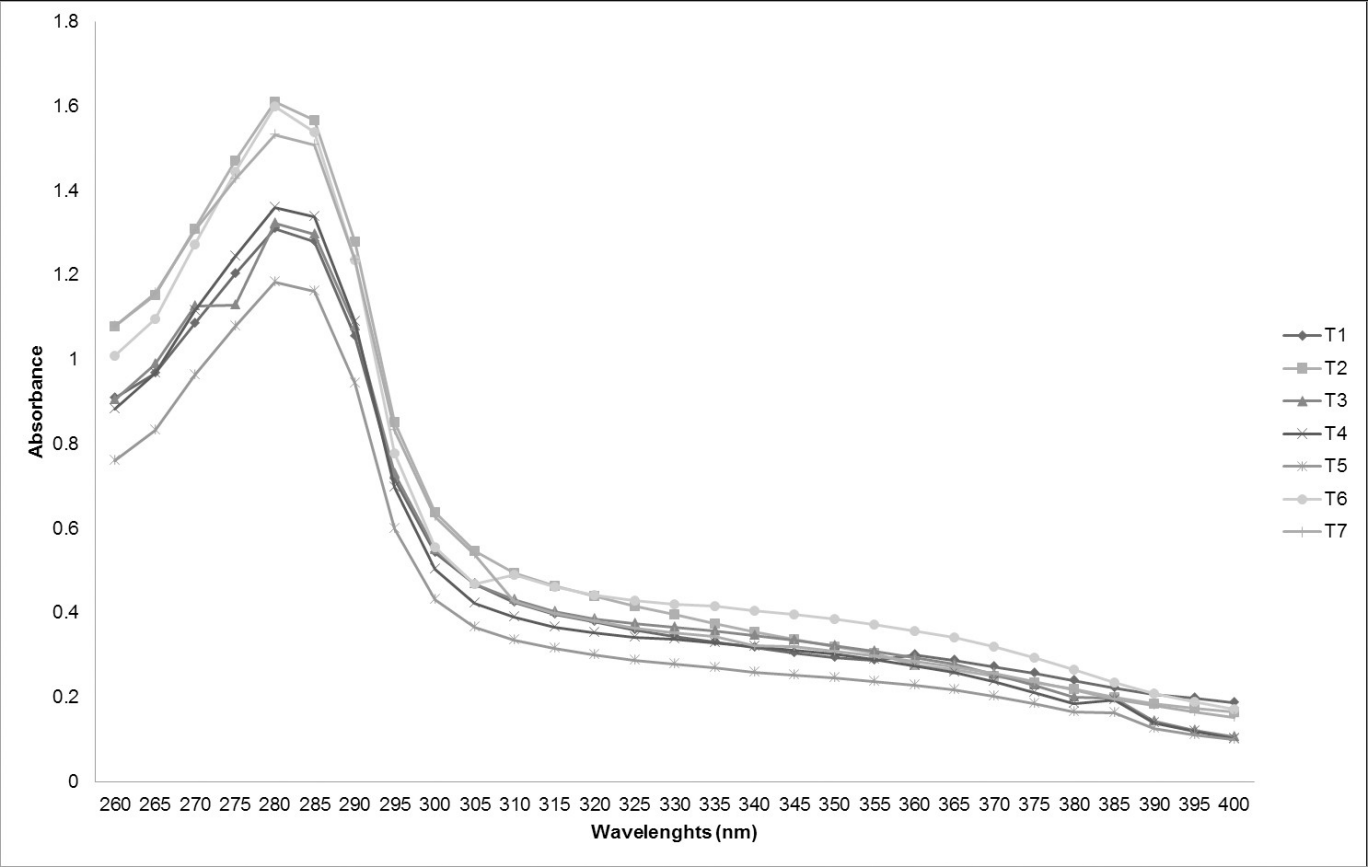
Absorption spectrum of UV wavelenghts (260 to 400 nm) to evaluate hydro-ethanol extract of *Dalbergia ecastaphyllum* (1000 μg/mL).

Among the phenolic compounds in plant species, flavonoids have been widely studied and are considered responsible for conferring protection against UV radiation [41]. These compounds show two absorption peaks between wavelengths 240–280 and 300–550 nm [42]. In this study, the formation of two characteristic peaks was not observed. The maximum absorption wavelength was at 280 nm for all samples of *D*. *ecastaphyllum* evaluated (Fig 1). Although the wavelength of maximum absorption was equal in all samples, the spectra differed between them in signal intensity. Differences in the spectral results show that the methodology is dependent on the profile of compounds in the extract and not only on total concentration. For instance, sample T6 had the highest flavonoid content, but presented an UV spectrum with intermediate absorbance intensity in relation to other samples.

**Fig 2:**
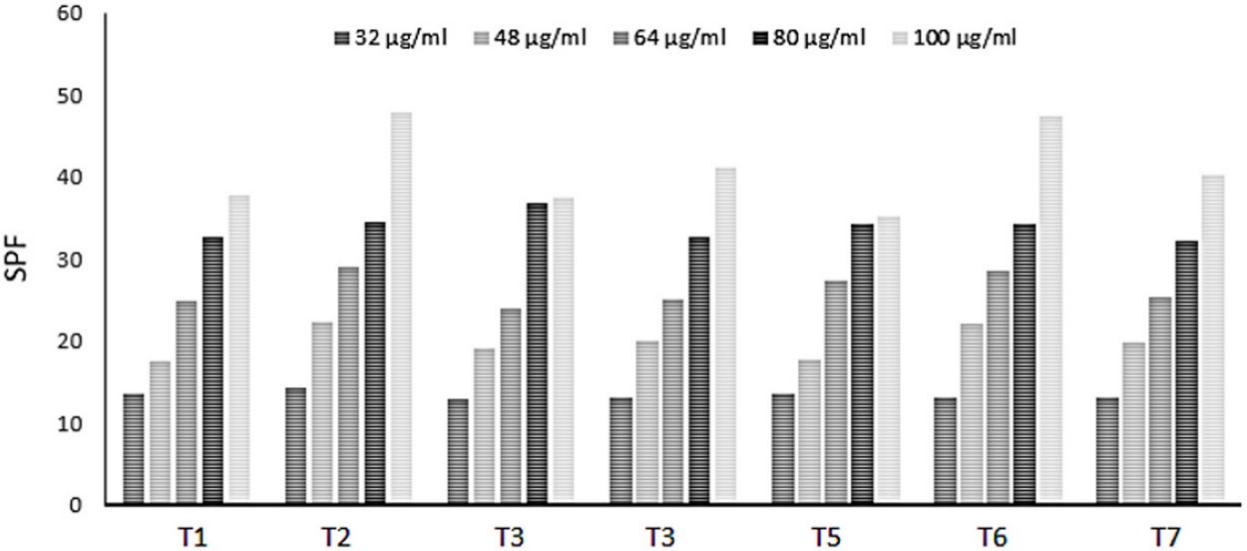
Sun protection factor (SPF) of hydro-ethanol extracts of *Dalbergia ecastaphyllum*.

The samples SPF values ranged from 13.08 to 47.80 μg/mL (Fig 2). The lowest concentration tested (32 μg/mL) in all samples were higher than the values required by legislation [43]. The results obtained for *D*. *ecastaphyllum* extracts also showed SPF values higher higher than those reported in studies with other plants. For instance, in ethanol extracts of *Marcetia taxifolia* (100 μg/mL), SPF was lower than 10 [44], in ethanol extracts of *Encholirium spectabile* (100 mg/L), SPF was below 8 [45]; and, in extracts of *Potentilla atrosanguinea* (120 μg/mL), SPF was 7.39 [46].

### Multivariate analyses

**Fig 3:**
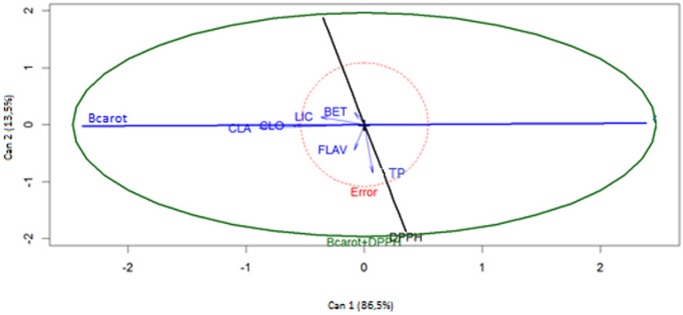
Canonical correlation analysis of phenolic compounds and methods of determining the antioxidant activity. CLA = chlorophyll *a*; CLO = chlorophyll *b*; LIC = lycopene; BET = β-carotene; TP = total phenols; FLAV = flavonoids; Bcarot = inhibition of β-carotene bleaching and DPPH = scavenging of free radical DPPH.

The canonical correlation analysis was performed to verify the relationship between the lipophilic and hydrophilic antioxidants and the antioxidant activity obtained by the two methods used. This procedure allows determining the strength of the relationship between two datasets and obtaining the canonical weights from the linear combination between those sets [47]. The analysis of the results in Fig 3 showed that canonical variables 1 and 2 (Can 1 and Can 2) explained 99.7% of total variation found. Can 1 (representing 86.5% of variation) showed a greater relationship with lipophilic compounds and the antioxidant activity determined by the method of inhibition of β-carotene bleaching. Can 2, which explained 13.5% of the total variation, seemed to be more dependent on the antioxidant activity evaluated by the DPPH test and on contents in flavonoids and phenolic compounds. These results allow inferring that the contents of flavonoids and total phenols are mainly responsible for the antioxidant activity determined by this method. But, it should again be noted that the overall results allowed to infer that the compounds profile present in the composition of each sample were the most determining factor in establishing the antioxidant action of extracts of the *D*. *ecastaphyllum* plant.

The canonical correlation and estimated canonical pairs between characteristics X (antioxidant compounds) and Y (antioxidant methods) in *D*. *ecastaphyllum* extracts are shown in Table 7. All the canonical correlations were statistically significant. This shows that characteristics X and Y are highly correlated, with canonical pairs (r) 0.95 and 0.75, respectively. The analysis of the variables in the first canonical pair showed a predominance of the inhibition method of β-carotene bleaching, while in the second canonical pair, the DPPH method was highlighted. The proportion of variance that is common for the two canonical variables, which is given by the canonical correlation coefficient square (r^2^), in the first and second canonical pair was 90% and 56%, respectively.

**Table 7:** Estimated canonical correlations between characteristics X (antioxidant compounds) and Y (antioxidant methods).

	Canonical pair	
**Variable**	1^0^	2^0^
BET	-0.08	0.17
LIC	-0.35	0.10
CLA	**-0.85**	-0.05
CLO	**-0.58**	-0.01
TP	0.07	**-0.64**
FLAV	-0.08	-0.34
Bcarot	**-0.93**	-0.11
DPPH	0.01	**-0.75**
R	0.95	0.75
F	7.81**	3.61*
r^2^	0.90	0.56
Α	0.0000067	0.0262620

CLA = chlorophyll *a*; CLO = chlorophyll *b*; LIC = lycopene; BET = β-carotene; TP = total phenols; FLAV = flavonoids; Bcarot = inhibition of β-carotene bleaching and DPPH = scavenging of free radical DPPH.

** significantly different (p < 0.01).

* significantly different (p < 0.05).

To check possible relations between the results for tyrosinase inhibition and the chemical parameters analyzed, Table 8 presents the correlation matrix of linear relationships between the variables considered, as well as, the values of significance. The values show a significant correlation between β-carotene and lycopene (r = 0.79), which is not surprising, since both are hydrocarbon constituents of antioxidant carotenoids.

**Table 8:** Correlation between variables in the multivariate analysis.

Variable	BET	LIC	CLA	CLO	FEN	FLAV	TIR
**BET**	1.00						
**LIC**	0.79*	1.00					
**CLA**	0.09	0.55	1.00				
**CLO**	0.01	0.46	0.84*	1.00			
**TP**	0.21	0.22	0.09	0.13	1.00		
**FLAV**	-0.59*	-0.45	0.19	0.28	0.30	1.00	
**TIR**	0.20	0.32	0.39	0.11	-0.39	-0.75*	1.00

CLA = chlorophyll *a*; CLO = chlorophyll *b*; LIC = lycopene; BET = β-carotene; TP = total phenols; FLAV = flavonoids; TIR = Tyrosinase inhibitory activity.

* significantly different (p < 0.05).

Moreover, chlorophylls *a* and *b* showed a significant correlation (r = 0.84), in fact, both are photosynthetic pigments. There was a significant negative correlation between β-carotene and flavonoids (r = -59). The correlation between inhibition of the tyrosinase activity and total flavonoids was negative and significant (r = -75), suggesting that the quantity of these compounds influence the activity of this enzyme.

**Fig 4:**
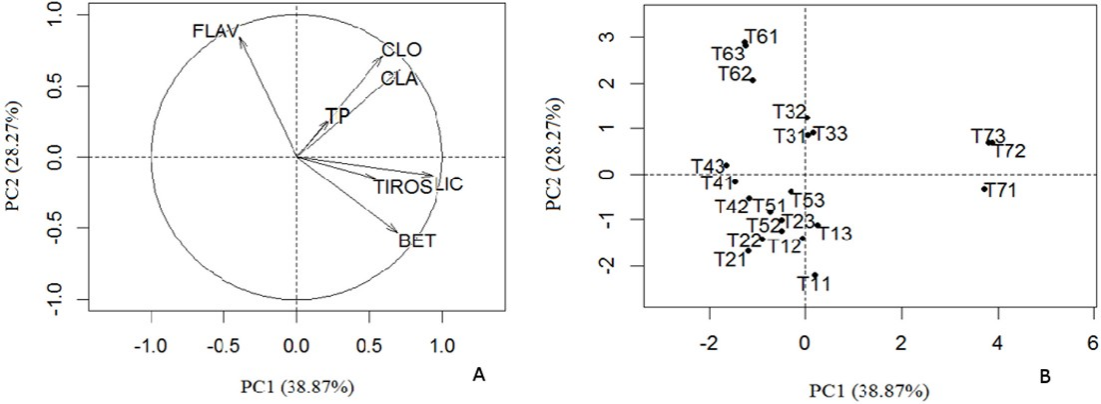
**Correlation of the dataset (A) and scores of the principal components (B).** Tyrosinase inhibitory activity (TIROS) and the phenolic content (CLA = chlorophyll *a*; CLO = chlorophyll *b*; LIC = lycopene; BET = β-carotene; TP = total phenols; FLAV = flavonoids) of hydroethanol extracts of *Dalbergia ecastaphyllum* (T1 = Itaparica; T2 = V. Cruz; T3 = N. Viçosa; T4 = Prado; T5 = Caravelas; T6 = Ilhéus e T7 = Canavieiras).

In this work, the PCA of antioxidants lipophilic, hydrophilic and tyrosinase inhibitory activity (Fig 4A and 4B) was also carried. The first two principal components (PCs) described 67.14% of total variation. Fig 4A shows the influence of chemical parameters in the new two-dimensional space established by the first two principal components. Fig 4B shows the natural distribution of the samples analyzed in terms of the chemical profile and properties. The compounds β-carotene, lycopene, chlorophylls *a* and *b* were characterized by PC1 (38.87%) with a significant correlation between the carotenoids and the tyrosinase inhibitory activity. The flavonoids and the phenol content were the variables that best contributed to PC2 (28.27%). The dispersion of samples according to the scores of PCs are represented in Fig 4B. Samples T3, T6 and T7 present different chemical characteristics and thus occur in isolation in the two-dimensional space. The remaining samples compose a group, which is indicative of samples with higher chemical similarity.

Sample T7 is characterized by the larger amount of lycopene and greater tyrosinase inhibition. Sample T3 showed, in general, intermediate values in the chemical composition and properties, while T6 displayed high levels in flavonoids. Samples T1, T2, T4 and T5 showed similarities characterized, in general, by low levels in chemical parameters analyzed. The results of this analysis are in agreement with those presented in Tables 2 and 3.

## Conclusion

Extracts of *Dalbergia ecastaphyllum* showed a high content of lipophilic and hydrophilic antioxidants and pronounced antioxidant capacity, evaluated by tests of DPPH and inhibition of β-carotene bleaching. All samples inhibited the tyrosinase activity and showed a good protective effect, suggesting the possible use of these extracts for protection against ultraviolet radiation. This is the first study that provides relevant information on phytochemicals in this species, indicating its potential for use in the cosmetic and pharmaceutical industries. However, further studies are necessary to identify chemical constituents of the plant, mechanisms of action and interaction between them.

## Supporting information

S1 Fig Geographic distribution where *Dalbergia ecastaphyllum* leaf samples were collected. (TIF)

S1 Table The UV absorption spectra of hydro-ethanol extracts of *Dalbergia ecastaphyllum* (1000 μg/mL). (DOCX)
