# Supplementary figures and images for: Antioxidant, photoprotective and inhibitory activity of tyrosinase in extracts of Dalbergia ecastaphyllum
Source: PLoS One. 2018 Nov 30;13(11):e0207510. doi: 10.1371/journal.pone.0207510 (PMC6269094; doi:10.1371/journal.pone.0207510)

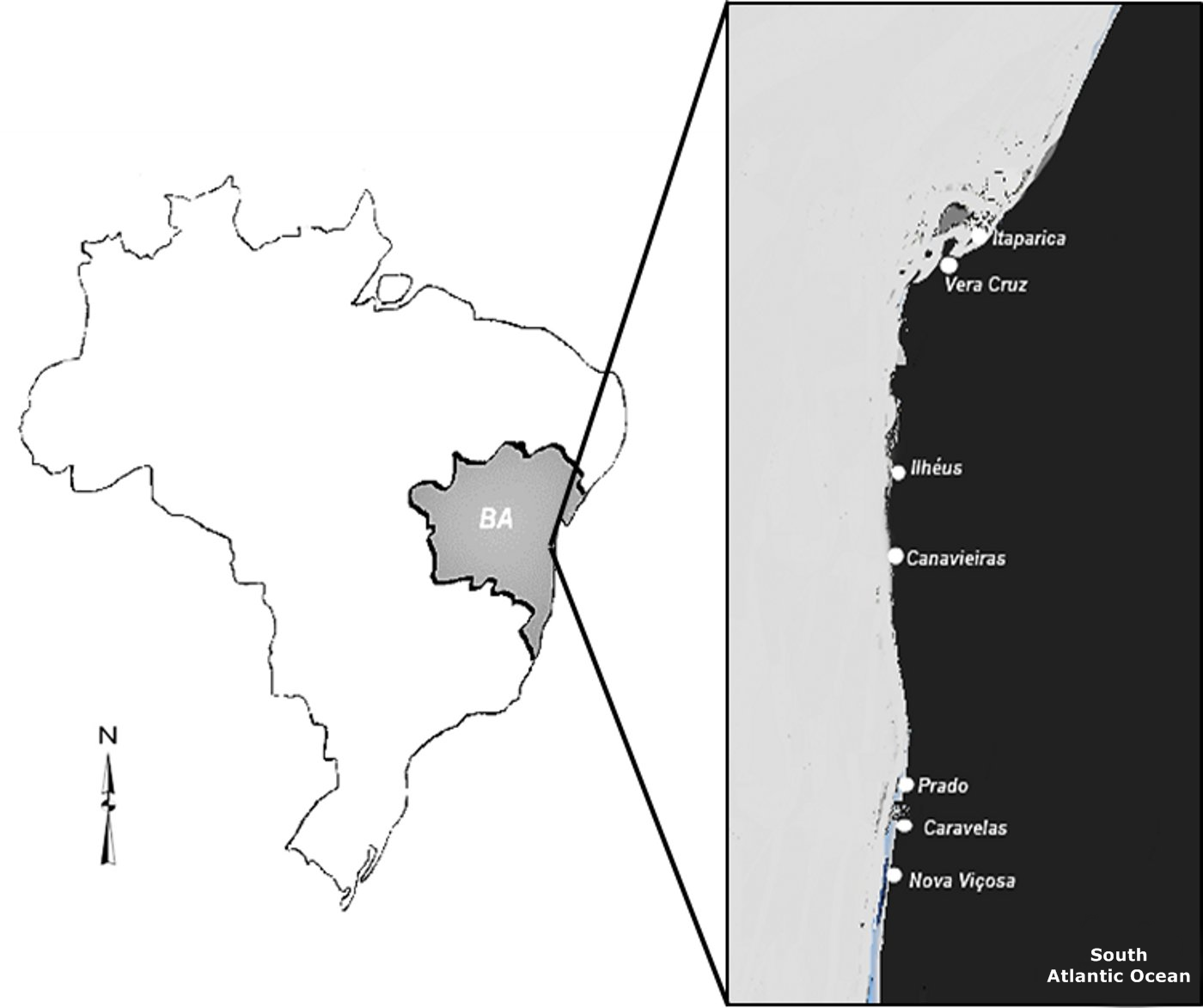

Supplement: S1 Fig — (TIF) [file pone.0207510.s001.tif]
