# Supplementary material for: Antioxidant, photoprotective and inhibitory activity of tyrosinase in extracts of Dalbergia ecastaphyllum
Source: PLoS One. 2018 Nov 30;13(11):e0207510. doi: 10.1371/journal.pone.0207510 (PMC6269094; doi:10.1371/journal.pone.0207510)
Supplement: S1 Table — (DOCX) [file pone.0207510.s002.docx]

S1 Table. The UV absorption spectra of hydro-ethanol extracts of *Dalbergia ecastaphyllum* (1000 μg/mL). T1= Itaparica; T2= Vera Cruz; T3= Nova Viçosa; T4= Prado; T5= Caravelas; T6= Ilhéus; T7 Canavieiras.

| **Wavelength (nm)** | **Treatments** | | | | | | |
| --- | --- | --- | --- | --- | --- | --- | --- |
|  | **T1** | **T2** | **T3** | **T4** | **T5** | **T6** | **T7** |
| 260 | 0.909 | 1.078 | 0.905 | 0.883 | 0.760 | 1.010 | 1.080 |
| 265 | 0.967 | 1.154 | 0.989 | 0.967 | 0.833 | 1.097 | 1.157 |
| 270 | 1.086 | 1.311 | 1.126 | 1.116 | 0.963 | 1.274 | 1.305 |
| 275 | 1.204 | 1.471 | 1.129 | 1.244 | 1.078 | 1.445 | 1.428 |
| 280 | 1.310 | 1.611 | 1.323 | 1.361 | 1.183 | 1.601 | 1.533 |
| 285 | 1.280 | 1.568 | 1.297 | 1.338 | 1.162 | 1.539 | 1.509 |
| 290 | 1.055 | 1.279 | 1.080 | 1.089 | 0.943 | 1.237 | 1.237 |
| 295 | 0.716 | 0.853 | 0.730 | 0.697 | 0.599 | 0.779 | 0.832 |
| 300 | 0.543 | 0.639 | 0.550 | 0.504 | 0.432 | 0.556 | 0.627 |
| 305 | 0.468 | 0.546 | 0.469 | 0.422 | 0.367 | 0.468 | 0.539 |
| 310 | 0.424 | 0.495 | 0.431 | 0.390 | 0.335 | 0.490 | 0.426 |
| 315 | 0.397 | 0.463 | 0.403 | 0.367 | 0.315 | 0.461 | 0.399 |
| 320 | 0.378 | 0.440 | 0.386 | 0.353 | 0.301 | 0.443 | 0.381 |
| 325 | 0.359 | 0.417 | 0.374 | 0.343 | 0.288 | 0.430 | 0.364 |
| 330 | 0.345 | 0.397 | 0.365 | 0.337 | 0.279 | 0.421 | 0.352 |
| 335 | 0.332 | 0.374 | 0.357 | 0.329 | 0.269 | 0.416 | 0.344 |
| 340 | 0.318 | 0.355 | 0.347 | 0.321 | 0.26 | 0.406 | 0.322 |
| 345 | 0.304 | 0.337 | 0.335 | 0.312 | 0.253 | 0.396 | 0.320 |
| 350 | 0.293 | 0.321 | 0.322 | 0.303 | 0.246 | 0.385 | 0.310 |
| 355 | 0.287 | 0.305 | 0.309 | 0.290 | 0.238 | 0.373 | 0.298 |
| 360 | 0.300 | 0.276 | 0.294 | 0.274 | 0.229 | 0.358 | 0.285 |
| 365 | 0.288 | 0.266 | 0.278 | 0.260 | 0.218 | 0.343 | 0.273 |
| 370 | 0.272 | 0.251 | 0.254 | 0.237 | 0.203 | 0.320 | 0.256 |
| 375 | 0.256 | 0.235 | 0.228 | 0.212 | 0.185 | 0.293 | 0.237 |
| 380 | 0.240 | 0.219 | 0.201 | 0.186 | 0.166 | 0.266 | 0.218 |
| 385 | 0.222 | 0.200 | 0.197 | 0.193 | 0.164 | 0.234 | 0.196 |
| 390 | 0.207 | 0.186 | 0.143 | 0.140 | 0.126 | 0.210 | 0.180 |
| 395 | 0.197 | 0.175 | 0.122 | 0.119 | 0.111 | 0.189 | 0.166 |
| 400 | 0.187 | 0.165 | 0.106 | 0.103 | 0.099 | 0.172 | 0.153 |
